# Clinical Holistic Medicine: The Case Story of Anna. II. Patient Diary as a Tool in Treatment

**DOI:** 10.1100/tsw.2006.334

**Published:** 2006-02-10

**Authors:** Sören Ventegodt, Birgitte Clausen, Joav Merrick

**Affiliations:** ^1^ Nordic School of Holistic Medicine, Copenhagen, Denmark; ^2^ Quality of Life Research Clinic, Teglgårdstræde 4-8, DK-1452 Copenhagen K, Denmark; ^3^ Quality of Life Research Center, Teglgårdstræde 4-8, DK-1452 Copenhagen K, Denmark; ^4^ Scandinavian Foundation for Holistic Medicine, Sandvika, Norway; ^5^ Vejlby Lokalcenter, Vejlby, Denmark; ^6^ National Institute of Child Health and Human Development, Jerusalem, Israel; ^7^ Center for Multidisciplinary Research in Aging, Faculty of Health Sciences, Ben Gurion University, Beer-Sheva, Israel; ^8^ Zusman Child Development Center, Faculty of Health Sciences, Ben Gurion University, Beer-Sheva, Israel; ^9^ Faculty of Health Sciences, Ben Gurion University of the Negev, Beer-Sheva, Israel; ^10^ Office of the Medical Director, Division for Mental Retardation Ministry of Social Affairs, Jerusalem, Israel

**Keywords:** quality of life, QOL, philosophy, human development, holistic medicine, public health, holistic health, holistic process theory, life mission theory, group therapy, incest, abuse, rape, sexual abuse, existential healing, existential (Antonovsky) coherence, Denmark

## Abstract

In spite of extreme childhood sexual and violent abuse, a 22-year-old young woman, Anna, healed during holistic existential therapy. New and highly confrontational therapeutic tools were developed and used to help this patient (like acceptance through touch and acupressure through the vagina). Her vulva and introitus were scarred from repeated brutal rape, as was the interior of her mouth. During therapy, these scars were gently contacted and the negative emotional contents released. The healing was in accordance with the advanced holistic medical toolbox that uses (1) love, (2) trust, (3) holding, and (4) helping the patient to process and integrate old traumas.The case story clearly revealed the philosophical adjustments that Anna made during treatment in response to the severe childhood abuse. These adjustments are demonstrated by her diary, where sentences contain both the feelings and thoughts of the painful present (the gestalt) at the time of the abuse, thus containing the essence of the traumas, making the repression of the painful emotions possible through the change in the patients philosophical perspective. Anna's case gives a unique insight into the process of traumatization (pathogenesis) and the process of healing (salutogenesis). At the end of the healing, Anna reconnected her existence to the outer world in a deep existential, suicidal crisis and faced her choice of life or death. She decided to live and, in this process, assumed existential responsibility, which made her able to step out of her mental disease. The advanced holistic toolbox seems to help patients heal even from the worst childhood abuse. In spite of the depth of the existential crisis, holistic existential therapy seems to support existential responsibility well and thus safe for the patients.

## INTRODUCTION

Many forms of therapy have been tried with rape and incest victims, but the therapy has often been less than effective and sometimes even counterproductive[1]. The more severe the abuse, the more difficult it has been to re-establish a normal emotional range and a positive philosophy of life. Victims have been treated with cognitive-behavioral and existential therapy, reality therapy, group therapy, and also with family therapy, analytical psychotherapy, supportive group therapy, and couple therapy[2,3,4,5,6,7,8,9, 10,11,12,13,14,15]. Even with these methods, it still seems to be extremely difficult to facilitate a process of existential, emotional, mental, and sexual healing that takes the patient all the way back to a normal state of mind. Quite surprisingly, we have seen this process accomplished where the patient was fully healed with the help of holistic existential therapy. The patient was so severely abused, both physically and sexually, that such a recovery was highly unexpected. We, therefore, felt the patient’s story worthy of publication, even though the patient diary is long.

Anna[16,17] was a 22-year-old patient who had been severely sexually abused most of her childhood by her father and two other men who blackmailed her father to let them have the girl for sexual exploitation by threatening to report him to the police if he refused. Her story is as terrible as they come and we have presented the case record[17], as well as her own patient diary, below to illustrate what it actually takes to get well again after such abuse. The most amazing thing about the story is Anna’s full recovery. She is now a successful university student with a boyfriend, today, several years after the treatment ended. It is suspected though that she recurrently will have to face philosophical, spiritual, emotional, and sexual problems in the years to come because of the abuse. We believe that her physical and mental problems have been solved. Only a strong and persistent relationship with her particular partner based on love, care, acceptance, and respect will give her the holding she will need in these difficult periods. Her challenge now is to use her knowledge from the therapy to build a satisfactory life.

We render Anna’s story in its full length, as it is important to show the huge, persistent cleansing work that had to be done by the patient herself in order to recover, when adopting the medicine of consciousness. It is particularly interesting to notice how many negative decisions have to be found and released before a severely existentially and mentally ill patient does indeed recover. The story also shows how the traumas available for integration in the therapy contained still bigger and more unbearable existential pains, as the patient gradually got more strength and more resources to go deeper into the “inner refuse bin”, drawing still nearer to live her own purpose of life. This is a very important sign of the patient’s existential healing. Her story starts just before she had her first breakthrough, recalling the sexual abuse. The relationship is between the patient and her physician [SV], together with the principal nurse [BC], and other nurses of the Research Clinic of Holistic Medicine in Copenhagen who gave both individual therapy[18] and holistic group therapy[19,20,21] that the patient attended in the start of her diary. This therapy is very much like the relationship between a caring father and a little daughter around 4 years old, due to the fact that she had a long course of individual therapy (see the case report[17]) and the abuse started when she was at that age. Please be warned that some parts of the story are very gruesome indeed.

As sexual assaults and rape are among the life events with the most dramatic negative effect on quality of life, the physician must take these traumas very seriously. In most of the world, sexual abuse of young woman is still very common and studies from different western countries indicate an incidence of about 15% of girls being assaulted sexually in childhood[2,22,23]. These patients are also more likely to be physically abused later by husbands and partners[22,24] and sometimes even the therapist who is supposed to heal and protect them. A reason for this is the sexual openness and the lack of normal sexual borders of these patients, making them easy targets for abuse, in combination with a high degree of suppressed emotional pain, making them highly projective.

These high numbers are disputed though. A problem with the sexually abused patient is that it is so difficult for the physician to be the one opening the old painful wounds of abuse because he will inevitably be the projection screen for some the worst things that happened to the patient. As the patient cannot contain the overwhelming emotional pain of the earlier events, it will be projected out on the surrounding world, and the therapist/physician will be the one most likely to get these projections. Any impurity of this kind in the therapist himself will then be dramatically energized and brought into experiential focus and without the strict professional ethics in this situation, it is practically impossible not to be “a part of the game”. When this is happening, one reasonable theory for explaining the high occurrence of “professional incest” seems to be that many therapists themselves have a background as victims of abuse. The rationale for “professional incest” comes from an unconscious longing for healing of the therapist’s own painful wounds on body and soul.

With a keen awareness of ethics and ethical behavior, allowing intimacy and not sexuality between the therapist and the patient with sufficient supervision, training, and therapeutic processing by the therapist, this problem can be handled[1,25,26,27,28]. In this case, a therapist who was abused him- or herself might be the most wise and helpful of therapists, as abuse fully integrated turns into a huge gift of understanding and acceptance of life in all aspects, even the darkest and most difficult. A therapist who does not know the evil side of mankind[29] is often not capable of helping patients treated as badly as Anna was. Only very few patients have an experience to the absurd degree of sexual violence that Anna had to recover from, and she did project very strongly, giving a good example of the above-mentioned problem.

The principles of existential holistic therapy and the process of holistic healing have been presented elsewhere[18,19] as the use of it on mental and sexually abused patients[26,30] and all these strategies were used here. Most of the tools in the advanced holistic medical toolbox have been used to heal Anna[27] and the difficult (level 8) technique of acupressure through the vagina and anus was partly developed for use in this case[28]. The core tools of the following process are found at the group level (level 5)[20,21].

The patient’s diary is a very important tool in holistic existential therapy. It allows the patient to confront all the things that appeared in the therapy, including the medical consultations. It also allows the patient to place the emotionally charged material in a safe place until it can be processed in the therapy. It also gives an important record of the process, so that track is kept of the often very extensive process of healing hundreds of gestalts. Most importantly, it allows the patient and the physician to identify negative sentences, the life-denying decision, from the gestalt, to help the patient integrate and let go of all negative beliefs and attitudes accumulated though the painful life events.

When reading the patient diary, it is important to understand that this text arises from a highly systematic and therapeutically well-supported exploration of the patient's subconscious material, with a strong focus on confronting the repressed negative feelings of the gestalt. The patient is encouraged to dive into these negative emotions and enhance them. As this is done, the old feelings of wanting to die, going insane or wanting to commit suicide appear in the patient's mind. These states of consciousness are not born out of the present moment, but out of the painful past, and thus they cannot and should not be avoided in the therapy. They must be understood as difficult, but necessary phases of the therapy. In the beginning we had the fear that the severe existential crisis described in the diary could actually some day lead to the suicide of the patient, which would make this kind of intense therapy unsafe for the patients. We have now closely observed hundreds of patients going though deep existential crisis, and quite remarkably, to our knowledge not a single one of these have ever tried to commit suicide, neither during nor after the therapy. This is in a way highly surprising, as psychiatric patients like Anna are known to often attempt suicide. The reason for the intensive holistic therapy being safe, in spite of the dark content of the stream of consciousness, seems to be that what really makes a person insane is not the content of the consciousness in the present now - we can dream the most horrible things and still be sane - but our level of existential responsibility. Choosing to confront the old traumas in existential holistic therapy in close contact with the therapist with the intent of healing, reflects this high degree of existential responsibility. So the patients can go really deep into their existential choices, and choose "to be or not to be", and still be completely sane, and therefore not at risk for committing suicide, because of the spontaneity of a psychosis.

There seems to be three fundamental themes of the existential crisis in the course of deep existential healing: 1) The loss of the old ego - the identity crises: Who am I? 2) Confronting the nature of the real self: what am I? and 3) Choosing life: Why am I here? What is my purpose and mission? The metaphor of the personal transformation, so well illustrated by Anna diary, is the transformation of the butterfly's larva: first it must loose its old self, to go into the transforming pupae; second it must remember its true nature and transform though this remembrance; and finally it must choose to be the butterfly, to get out of the pupae into the world. Please notice that Anna became a student of holistic medicine, and her unique talents of understanding the process of healing and her own needs in the therapy was an important reason for the development and use of new tools like "acceptance through touch", and "acupressure through the vagina"[26,27,28].

## DIARY OF ANNA

**Monday of the group course**

Yesterday evening Søren (SV) arrived when nearly all of us were sitting at the fireplace. I was very insecure. I was just thinking of how afraid of everything I was and how much I wanted to go home, as quickly as possible. Søren came over to me and was extremely considerate, most affectionate and nice to me. He hugged me a lot and said warm words.

Evening. Already this first day I feel it as a hundred days. Søren appears to be at his best today, and I have been glad to be here. Today we should get to know each other further, shake hands, hugs and a few words; and I noticed all the other participants. Actually, there are many lovely people here, and even those whom yesterday I thought would tire me, I do contain.

Afterwards we should choose a partner. A young man, Peter, came over to me and told he felt kindness towards me. At first he scared me; then Søren comes over to help me to sense, and yes, I felt kindness towards Peter too. So we became partners. Later we made exercises where we showed each other attention in turn, care and respect. I think Peter and I were doing some fine exercises – and now I am very glad that he is my partner. He seems very affectionate and nice.

Later on that day my abdominal pains and shoulder pains worsened, and I got headache. Søren helped me and backed me in giving myself away to Peter and not being afraid to both give and receive physical care; thus we talked about trust.

Peter and I talked about wanting to break through, finding out what we needed, and making sure to get our needs fulfilled. I am quite convinced this is the way it will turn out. I so fervently wanted that, although I try to hold back, to be the nice girl, and therefore I hide the fear in my abdomen. I want, indeed!

**Tuesday**

I work at finding negative resolutions:
*I trouble other people*
*I am troublesome*
*I am a burden*
*I am impossible*
*It's my fault*
*I am not good enough*

**Wednesday**

It's in the afternoon and I am sitting at the fireplace together with Søren. I tell him that I am frustrated and scared as well as that *I don't deserve to live* and that *I don't deserve life*. Søren takes me on his knee and let me nestle close to him like a little child. I tell him that *I have timedout*. We talk a little about containing the feelings, and what strikes me isn't so much what Søren is telling, but it's the nearness, his holding me close and containing me, while I didn't thought I deserved to live; and there I was sitting on his knee and got slowly better.

Later the same day, in the evening, we are a few from the center, who have gone to the beach. Last only Søren and I are left. I feel sad, as I have started remembering ever more difficult episodes from my childhood, all with the topics of abuse. A fire is lit, and I am lying on a rug, while Søren is sitting besides me. He asks me to go into my feelings. His presence and attention, which seem one hundred percent directed towards me, makes me calm, and I am not afraid to go into the feelings; I feel confident in his company. Furthermore, I feel such a trust in Søren, which I have never experienced in any person before, and therefore it is very important to me that we are alone as I am a very private person.

So I was lying there on the rug near the fire and I experienced how my body began to shake, almost in jerks, and how these jerks increased in intensity and how the intervals between them became shorter and shorter. Along the way Søren is meta-communicating and tells me that I shake, because of the energies, which my gestalts have bound and now are in the process of releasing.

I tell Søren in detail, which episodes are coming up from my sub-consciousness, and we have got many pauses en route, which I experience as most tactful by Søren. It should thereby be possible for me to come into something very profound and difficult. The sentence "*this is unreal*" comes up. Now I experienced, while telling Søren what I saw and sensed, that I spoke with a mechanic, strange voice, and I realized that the voice was not mine. Parallel to this, I experienced that I was standing at the end of the fire and was undergoing all those things, I was telling about mechanically while laying down. In other words, standing I sensed the feelings and the pain connected with the abuses and infringements, and laying down my framed body talked about them.

This experience was real and very "physical"; I could distinctly feel that I was shifting between the two bodies. When life was hurting it got unreal to me and thereby I was able to hold on being alive again. Down on the beach I let go of the sentence "this is unreal". It was like having received my first revelation.

Søren told me, after we have worked, that previously I was borderline which makes me terribly sad. It aches so much inside myself; and to think that I should be psychically ill! His stressing that I am ex-borderline now helps. He holds me close, gives me some of his warmth, and I can feel that he cares for me although I am a strange one, and it feels indescribably nice and at the same time almost incomprehensible to me.

Here are some episodes from the last few days I have come to think of. Spankings: My father often punished me by giving me a spanking on my naked behind. Once I was spanked, because I was eating too slowly (a dish with chops and tomatoes); as I was drawing with my green lettering pen outside the paper onto my blue velour track suit. Sometimes I got spanked if I did not eat quickly enough at supper and then again after having sat at the table for three hours. Actually, it was accidental whether I got spanked for not eating fast enough. I guess it depended on my father's mood.

Communication: For days my father could be angry and not say a single word to us, and we knew that in any case we should not say a word to him either, nor should we do anything wrong at all. However, the problem here was that I never did know what was wrong, because there was never something for sure. If he needed to beat or yell, he just did so. Beaten and yelled at, I was never told why. Therefore, there was never anything for sure because there was never an explanation why he did as he did. I dreaded by father, and was mortally afraid of him and his violent changes of mood. After being beaten I was always sent to my room and was only allowed to come out, when he told so. There I would cry, silently, so he should not hear me.

Constipation: As a baby I ate the foam-rubber mattress in my cot, toilet paper in the bathroom and paper from the newspaper if accessible. Later on I very often got constipation and I remember that last I got it at the age of 7–8 years, when my mother had to "dig the shit out".

**Thursday**

I have got a new life and a new face. I can easily sense how terrified I am. Talked with my partner Peter about it. We had become interdependent. I went into him because I am not able to be in myself; this is unreal. By going into Peter I get out of reality and so I am timed out. It was so tough for me when timing out as a little child.

*I am empty*
*I am hollow*
*I want to go away*
*I want to live* (14 days old: I fight for living, I am in trouble and through my will, I do survive)

**Friday**

Today I have had quite an experience. A real, beautiful and loving experience with Nete. All my kindness went to her and it was reciprocated tenfold by her. I was blissful. Never before have I experienced wanting to give love with so much devotion; it made my head swim. I am eternally grateful to Søren for having arranged this totally unique and fantastic love background, which I feel we have got here at the course, where we are giving and receiving love freely and without any restraint, either in the form of words, physical care or in the mind.

It was during this totally fantastic and very beautiful experience that I got a full comprehension of many aspects of Søren's holistic medicine. I was deeply moved and am indescribably happy I had testified that love can be the door to a genuine meeting between two persons, who were so far away from each other to start with.

I can sense that love is constantly flowing here, and I myself have never believed that I should experience so much warmth and kindness by other people. I have started believing much more in love as a medicine, and I can feel this gets myself closer to the trustfulness, and actually this is a much greater gift to me. And how could it possibly be different? Søren is the first person that I have really trusted, i.e. pure trust, so of course he is also the one who opens me for that trust in other people as well. This is one of the greatest gifts I can get: to regain the trust in other people!

**Sunday**

At a time I choose to lie down as my stomach ached strongly, much more than usually, and Søren searched on my stomach for the place aching. He found the place, pressed at the spot, and then I felt the pain; a lot of pain. I cried violently and did really manifest myself. Søren asked me various things, while touching that place on my stomach, but I was not able to reply to his questions. The convulsions/jerks I had experienced on the beach together with Søren started again, and soon they turned worse than that night. I got cramps in my head and in my legs as well, and the cramps were more and more intense. Now Søren supported my head, and I began to feel a burning pain coming from the inside of my head. It ached so much that it felt like an imminent explosion. Søren told me that these were fever cramps from when I was a little baby and from which I almost died, my mother and father not being there for me. I liked that Søren was meta-communicating meanwhile. It reassured me in the situation.

The cramps grow even more intense, and soon I am beating myself, exactly like at the time my brother and I played when we were younger (a play where I pretended to be mentally handicapped). It grew ever more intense, I shake all altogether, and it was completely wild. I suffer unresistingly. Then the sentence "*I want to live*" turned up, the sentence being thus connected with this situation, this gestalt.

I almost screamed the sentence, and my voice reflected huge and intense pain. On having said the sentence, I got relieved. Soon bliss itself washed over me; never before had every single fibre been relaxed so much. Afterwards I released the sentence "I want to live", and again I shivered a little. Harsh.

Søren hugged me, and I can feel he is there for me; no matter what. I am so blissful! This is my second revelation. At the time I decided I wanted to live, I did very nearly die. I am surviving exclusively based on my will, and ever since have I been feeling as if I did nearly die. I have not been living, but did very nearly die. Release – bliss. One gestalt poorer.

**Monday**

In the evening I am manifesting myself again. I am in my room. Something big is underway. As many participants of the course are manifesting themselves, Søren has to leave me at regular intervals. This I am able to contain, and I am constantly feeling he is together with me in the mind; the mere idea of this preparing myself to pull through.

The manifestation initiates where I am in my mother's womb. I move a lot, but I have the impression that she does not want to feel me. So I move ever more. My body shivers; writhes and soon I fling body and head backwards and forwards. Søren back me all along and ask what is happening and what I am feeling. I feel his care for me, which makes me going direct into the pain.

I manage to get all the way around my mother's womb, I rotate, and soon I begin to feel a sort of rope round my arms and chest; I got wrapped up in the umbilical cord. I get the impression I should split; the umbilical cord feels like a straitjacket. I get water, amniotic fluid, in the throat, and I cannot swallow it. I get strangulation sensations. However, my mother still cannot feel me, she does not want to feel me, and I decide *NEVER MIND*. Soon afterwards my body become quiet and something else turns up.

I am back lying with my baby brother. My mother and her new partner have sex, and I say "*She does not want to*." Now I am on the track of the sexual assaults. After having said "She does not want to", I now hear and see the arousal and say: *I DON'T WANT TO HEAR IT – OVER*. Then comes: *I DON'T HEAR IT*. I start saying *THIS IS BAD*, repeat it many times and do not really want to proceed. I am strongly maintaining that THIS IS BAD, and Søren tries in different ways to proceed. He is very tactful and extremely delicate in the situation and handles my situation so gently. Now comes the sentence *I DON'T WANT TO KNOW IT*, *then I DON'T WANT TO SAY IT*, and this while I am crying and incredibly frightened. Søren back me up and let me know that he is taking good care of me. Then comes the sentence *IT'S NOT MY FAULT*, and here I can sense that I mean it is not my fault that I am arousing him (the perpetrator) and that the event is taking place. Then comes the sentence *THIS ISN'T OK*, and here I am uncovering it, I guess. Then comes the sentence *THIS IS SECRET* and the sentence *YOU SHOULD JUST KNOW THAT YOU WILL BE PUNISHED, HARDER THAN EVER, IF YOU TELL IT.*

Again Søren try to encourage me to proceed and to go deeper into the episode. I trust Søren one hundred percent, and I know he can help me and he wants to back me up through anything tough. Now I sense that the door is being opened, and I can hear the noise of somebody trying not to make a sound. It is my father coming in. He sits down on the edge of my bed, hold of my waist and place me, my back turned, onto his penis. He hives me up and down over him, has an orgasm, and lay me back in all the blood and sperm. While this take place and during most of the process, my body make twitching movements, in jolts, like during an intercourse.

Then comes the sentence *IT'S NOT ME*, and I say this because here I go outside myself and fly, I jump from an aeroplane and float in the sky, but I know I will hit the soil again. I am floating painlessly and land on the soil with a crash. Then come the sentences *I DON'T WANT TO* and then *IT IS MY FAULT*, and then *YOU WILL GET PUNISHED*, and *I DON'T WANT TO HEAR IT*.

While this happens I am back, 4 year old, talking with a very mechanical voice, almost in twitches, and the voice only disappear the following day in the afternoon.

Several things come to me as memories the next day: How my father had punished me, because I tempted him. How my hair was cut, how I was dressed and brought up like a boy to reduce this temptation. How I carried on with this, when I myself started buying clothes: men's clothes, businessman shoes and the like. I looked like a grown-up man in my clothes. How also my mother was feeling guilt; therefore she shut herself into the bedroom, unable to take care of me and love me, even when I tried to encourage her. How finally I had to leave the bedroom, because I could feel she hated me. Maybe she punished me by her ill treatment; maybe it was her payback?

The food. How I was not allowed to eat what was supposed to be my father's filling for his packed lunch. Therefore, I ate roasted onions, macaroons and raisins for lunch after school. I was hungry, but there was not any food for me.

**Tuesday**

I still talk mechanically, am a little 4 years old girl who is very scared of other people, whom I believe will punish me, every single one of them. I feel as if I am in day care now, where everyone will be after me. Søren is incredibly nice and endlessly affectionate towards me. He is like, often before, my father, however it is completely different this time. Now I feel he is the only one, who really can protect me from all the evil people, and I prefer to stay near him. At breakfast he makes funny puppet dances with his fingers, which make me laugh a lot. I feel how I am only 4 years old and I am really not older. Søren's dancing like that with his fingers make me happy and warm inside; he meets me as the 4 years old girl I am, and he does it with consideration and affection.

Later down in the hall I do not dare at all to look at any of the other participants, and I am sitting safely besides Søren. He asks them, at my request, to take good care of me and let me be alone for a while. After a short while the twitches, the intercourse movements reappear. I am sitting beside Søren, I am totally confident because of his presence and I just go into the feeling. I suffer unresistingly. Now I do not care whether the hall is full, whether everybody is watching; because the hall is full and yet it is empty. Only trust and care are here. I do dare, and therefore I suffer unresistingly. The movements become more hefty and powerful. I can feel it is happening/I am getting raped many times indeed. Just as I am sitting there I get ever younger, and at the end I have got no language. Am I 3 years? 2 years?

The following sentences turn up*: I CAN DO NOTHING, IT'S MY FAULT, I DON'T WANT TO, and I CAN'T STAY ANYWHERE.* Together with Søren I return to the episode, where my dad had sex with me and afterwards he turns me round so he can spank my behind: everything is wet in the bed, and my whole body is aching, and I cannot be in the bed. I can stay nowhere and in the morning my mum clean up.

**Thursday**

Yesterday everyone from the male group found their purpose of life, and we had a wonderful evening together on the beach. We from the female group had arranged entertainment, including belly dance and songs, food including coffee, wine, chips and cake, and then we served them. Our mantra during this was: no control, no criticism and no claims. Well, I must admit that actually I enjoyed a lot just being allowed to do anything so they should feel comfortable. And they were much greater, when meeting us there at midnight; they were bloody men, indeed!

So this morning we should find the female's purpose of life. While meditating to music, the men went about whispering gifts to us women. At a point Søren come to me and whisper in my ear something basic about my purpose of life. I got so touched that I started weeping. It felt like so incredibly great words, and I can hardly believe that the words he told were about me.

**Friday**

I find my purpose of life: "I bring warmth and joy". I find this as the finest and most valuable gift I have ever got.

**Tuesday**

Sunday I came home after two fantastic weeks at the summer course. Yesterday I was 11 years old and wished for a fancy pink school bag. I am in flow, it feels so nice. Today I have turned 12 and have got the first period. It was a mixture of relief (I am like the others), sadness (because I should have it for the next 40 years) as well as a dislike about growing up. Ironically, as at the age of 12 I was already a little adult. I played a lot with my doll I had bought and took it for an evening walk. I felt I was back to the time, when I played with my big dolls house, something I was completely crazy about. Today I wish for a doll's house of my own. Again I think of the nice words Søren whispered in my ear about my purpose of life. It warms.

**Wednesday**

Today I am 13 years and wish for a dress.

**Thursday**

Good night 14 years and still without the dress. Sigh.

**Saturday**

I dream a lot for the time being, and Thursday night had a particular dream. I dreamed that I confronted my dad with the sexual assaults, that in the dream he got scared, and that the accusation was exact. He could not really answer back. Heavy artillery indeed I had been driving in position.

I was at a really nice concert, and at long last I had bought a super fancy dress, which I was wearing, and I felt simply so dishy! I felt smashing indeed, and it was really a fantastic sensation. Hurray!

Later I went dancing in the nightclub. It was divine and even a little better than usual. I did it to my heart's content, and it was fantastic!

**Medical consultation at the clinic run by Søren (Quality of Life Research Clinic)**

Ever since the assaults I have regularly reverted to them by myself, on the hard bathroom floor, where I at the age of 7 years made quick and hard masturbating movements with three fingers, which took me back to the rape. I reverted because, although this was foolish, it was a form of contact between my father and me, the only contact we had besides when he hit/punished me.

I also did revert when having sex with my boyfriend. The brutality/fierceness was nearly always part of it, as I would prefer to be taken from behind quickly and hard, be pinched and bitten on my nipples.

Søren helped me to feel first the pain in my vagina aperture, my uterus, then my whole inner pelvis. He contacted the scars in my vulva and vagina; tactfully, balancing on a knife-edge. (The techniques of holistic pelvic examination, acceptance through touch and vaginal acupressure was partly invented to be able to heal the existential, mental, emotional and genital scars of this patient[26,27,28,31]). He brought me home to my uterus. He had been healing something inside me; this morning I saw my body as rounder, smoother and much more feminine. My psychosexual development is no longer like that of a four year old. I am on a fair way to become a woman. I got an enormous gift from him, and I am very grateful to him.

Søren dealt with healing the scar on my right labia, which was continuously chapping. It got better while he was healing it and en route the pain was very close to unbearable. At first came the physical pain, which was almost unbearable and then I enter into the emotional pain. There is a huge and intense gestalt, and my hands are completely clenched. I feel an enormous rage and can also feel that I am not able to release the rage although the sentence "*NOW IT'S DAMMED ENOUGH*" is swimming in my head like a mantra. Afterwards I get in dialogue with my inner little girl after having found the sentence "*I HIDE*"; thus I have been hiding in my own hand, when getting abused. It prevents an adult life, where I can react and be present in myself. I let go of this sentence, and now the rage changes. By the way I had a splitting talk with that little girl. As Søren asked me to release the sentence she tells me: *YOU DON'T KNOW WHAT YOU ARE DOING* and *IT'S DANGEROUS*. Then I tell her that we cannot play hiding the rest of our lives and be lying under a blanket without saying anything and without moving the rest of our lives. We must live instead, I tell her; thus I am able to release.

Afterwards the rage can get out, and at first I chat, then hit, then beat and hammer with clenched fists on two pillows, while I am saying: "Now it's dammed enough!" That helps. It's nice to let go of the rage, and that ease me a lot.

**Sunday**

New negative sentences:
*I am ugly*
*I am afraid of men*
*I feel sorry for myself*
*She is too much*
*I am getting punished*

**Saturday**

Now I realize that my dad did often assault me in the morning before my mum was awake.

**Wednesday**

The sentences:
*I don't need you*
*I need nobody*
*There is nothing to come after*

turned up in connection with my getting stock of something, a gestalt, in which I suffer and which makes me very sad. I am talking with Søren, and more than anything else I need affection and care, I distinctly feel that, and still I do not need anyone/you. It was so obvious that what I needed most was somebody. Then appeared the sentences. And the reason why I do not need anyone/you is that there is nothing to come after. Nevertheless, I am not able to get anything.

**Thursday**
Over the breakfast I phone and talk with Søren about how I am doing. We talk about my sentences, and he says to me that he is there for me if I should need him. I just have to tell him I am need him. This is a good and extremely important thing for me to learn; it is a way of letting other people in. It is a way of approaching others. Presence. This opening is primarily due to Søren, as I feel I may "practise" on him. I can practise needing him and then learn to contain either his being there that very moment or having to wait for a better opportunity. It is safe to practise with Søren.

**Tuesday**
The sentence *I AM IMPOSSIBLE* turns up.

**Wednesday**
Two sentences appear:
*IT'S ABSURD*
*WHEN I COME, I AM DYING*
New memories came to the surface after that; about my aunt's treatment of me during the summer holidays at her place. I did everything wrong. I was always in the way. And about my father: I am seven years. The night is a hell. I do not sleep at all. I remember how I simply did not dare to sleep on my back, when I was younger. When my dad came into my room at night to have sex with me, I was always sleeping on my stomach. And when he had finished with me, "his screw doll", I would always return lying on my stomach, in the blood and sperm, and pull my arms completely under by body trying to give myself a little care. I almost see the whole episode like in a movie; how he sneaks up onto my room, gets hold of my waist, takes off my pyjamas trousers and briefs, and places me down over his stiff penis, my face turned off him. He moves me like it pleases him, all the while my small doll legs are kicking. He does not care whether I am completely dry, whether he gets crooked up inside me, whether I am suffering, whether skin may be trapped, whether I am bleeding, whether I get cuts and wounds. Sometimes he also laid me on his thighs taking me up and down over his penis.

**Medical consultation at the clinic run by Søren (Quality of Life Research Clinic)**
I have been bleeding a little from the intestines for a while. Søren examines my rectum. I have the impression of getting filled up two hundred percent and it is tremendously uncomfortable for me. It feels as if a refrigerator is crammed into me, and I say *I CANNOT STAND HAVING IT INSIDE ME*. I do not feel there is any room. It turns ever more unpleasant, worse and worse. I would like to run away screaming. I cannot be there at all; it gets worse and worse. *I CANNOT FIND MY BEARINGS AT ALL,* I said, and now I am in the space with planets passing and stars hanging and shining. And I want to go home. I feel there is something completely wrong and that I am not myself. Now the disorientation takes over completely, and I am totally gone on. I do not know at all where I am. I would like to say to Søren, he shall be careful not to become like them (my dad and mum), but I cannot get myself to tell it. I say, "This is the worst thing you have done to me".

Afterwards I apologize for my projection. I cannot accept him in any way at all. I think "*HE IS A PIG, HE DISGUSTS ME*" and at the same time I am awfully ashamed of myself. The dislike is too heavy, the shame too overwhelming; I am not in me, I am not at home. I can feel I am beside myself, and this scares me terribly. I cannot find my bearings at all. Shame and pain. *HE SCARES ME, HE IS LIKE THEM*, I think. Søren says that the pain I am experiencing now is too intense for me to contain, and therefore I am projecting it onto him. I know he is right. I know I am projecting big time onto him. I am not able to have it inside me; I feel I cannot contain it, not at all. The following sentences come: *I am hiding, I am in the space, he is disgusting, I am cooping up, I don't care, he doesn't care, there isn't any room for it*.

I feel bony and thin, skeleton-like. Søren asks whether I was thin as a child. At first bun fat, then thin, I answer. At our place there was actually no food. I often ate roasted onions or half a bag of macaroons, when coming home from school. Usually there was not any real food for me. The fillings available were for my dad's packed lunch; the rye bread too, so I was not allowed to take from it, or one slice at the most so nobody could see that I have taken some. Søren says lack of care, and while he is saying the word I chant in my head: *I DON'T CARE*.

I stay at home from work. We get hold of Marianne (the nurse) and asked her to assist in conveying me holding. She would like to. I am just sitting in Marianne's arms while we are listening to some nice music. I almost fall asleep there in her arms. As Søren arrive we deal with my time line. Marianne wrote down everything that did happen.

## The Notes by the Nurse about Anna

Anna is back in time, about 2–3 years old. They should remove her nappy. She could not really speak yet. Both her mother and father did harass her; the mother abused her violently, her father sexually. The father was the one doing it at night, while the others were sleeping or in the morning when her mother was still sleeping. He did not care if there was blood on the sheet. Anna experience that all this fills her up, it fills her whole stomach. The mother used a spoon in order to make her bleed, so her father should not abuse her. Anna experienced that the mother did it to be nice to her and to protect her from the father. She has got the feeling that her sister was been abused as well.

The contents of the gestalts*: She (the mother) hates me. He (the father) hates me. My sister hates me.*

The mother did not want to give Anna prunes, as asked for by the father, when Anna was constipated. They called her a particular pet name, when carrying out the ill treatments. When calling her Anna they could not do that. It was as if the mother and the father knew what the other was doing. Anna also thought there were other people who knew something about what was going on. Many different people had baby-sat her. They should have seen there was something wrong, when changing her. The father stopped, when she was about to enter school, but the mother carried on and Anna remembered how the mother laid her on the changing table as she was 7–8 years old. At that moment Anna wished to die. Was sent outside, did smash a rough plate hoping she could cut her wrists. She also tried to run away and hide. The sentence *I HIDE*. The little hand is completely squeezed and unapproachable. Extremely slowly Anna opens her hand again.

*Signed by Marianne (the nurse)*

**Monday**

I talked by phone with Søren about revenge and rage. I am totally, terribly angry. I feel as if my rage stretches thousands of kilometres; really far! First I do not really want revenge, but then the vindictiveness comes to me. I say a lot of angry and confronting things to my father at that moment, and as soon as I have said them, my back is feeling better.

**Thursday**
I read in the newspaper today that a 53 years old father, who received 2 or 3 years of imprisonment and paid twice DKK 35,000 damages to his two daughters, that he had abused over a four year period. The girls were 7 and 12 years old. [The legal issue was discussed many times with the patient during the therapy; the focus of discussion was the best interest of the patient. We agreed that she was not strong enough to confront her offenders in the court. After the therapy she could go to court, if she wanted to From a holistic medical perspective the patient's interest is above the general interest of society; justice is less important than the health and well being of the patient].

**Sunday**
**Medical consultation at the clinic (Quality of Life Research Clinic) with Søren and Marianne, the nurse**
As Søren and Marianne arrived I was in the paranoid psychotic condition. [It is important to notice that this psychotic episode seemed to be a natural part of her healing and she was not drugged during the days of mental crisis]. The first time I noticed that I was paranoid was on Friday. I was walking in town shopping and I got scared on the road. It is difficult for me to find the words for it, but I was aware that I was getting hot flushes, becoming dizzy, nausea and a huge desire to run home. In fact, this happened again the following three days when out for a walk. I get afraid when people walked in groups. I was especially scared of men on the road; I thought they wanted to snatch me and take me along. I thought they were able to jump inside me, like invading me and taking what is mine and in a way occupying me. I thought there was nothing I could do and they could do whatever they wanted, for instance violate me, beat me and rape me. They might pour boiling water and ice water into my vagina, stuff tins up inside me and tie me.

The rest of Saturday I did not go out. Again panic after having been outside for a short while; I could barely take the receipt from the man, who brought me a pizza. This was to be too close to him. I feared he should catch my whole arm and just drag me along. Again things are running about, I got hot flushes and feel terribly unwell. I feel almost as if I am chocking. That sensation I got earlier today as well, when a friend, Peter, called. We were eating so I told him I would call him back, but as I had put the receiver down everything in me shivered and trembled, and I told my friend they now could also come in through the telephone. I felt invasion, thought there was no refuge for me; that they could come and hurt me also through the telephone, as if Peter could actually come out through the telephone and be physically in my room.

As Søren and Marianne came, Søren made in clear to me that if I was not willing to put the lid on my "inner waste bin", if not now then after having processed, he would have to get in touch with a psychiatrist, a good friend of his. This makd me completely terrified, and I got terribly afraid he was going to do that and that they will fill me with drugs. Through our conversation I realized that Søren could not have me walking about being psychotic, because I am his responsibility. Furthermore, I saw how I have neglected my own responsibility in this situation. I have continuously carried on opening forever more stuff although I had not managed to come to terms with and integrate well what I had just been processing. I behaved like a too eager little girl who, because I got unlimited love when working with tough things, just unrestrainedly kept on opening new and more hideous things. I realized that it was connected like this, and then we proceeded.

Next Søren dealt with healing the scars inside my mouth [the standard procedure described in[32], similar to healing genital scars described above]. First he touched on the left side of my mouth. I cannot feel anything at first; some time passes by, and then I begin to cry. I tell how much I have been thinking of my paternal uncle the last few days. I have always had an image of him as most arrogant, evil, indifferent and indescribably cold; terribly cold. I feel an indignation/rage towards him, while I am lying here; he did always feel above all of us, and in fact he is just so small, such a small shit. While Søren is dealing with the scars, more and more images come to me. They concern my paternal uncle, who had raped me totally five times. I shall give a more detailed description of them tomorrow. The episodes in question are particularly connected with the scar I have got in my lower mouth, which is so striking that its surface almost feels like a tooth. As we are dealing with it, I feel like going to scream, and we try this in the duvet. It's as if I don't know at all what screaming is about. How do you scream, I ask Søren and Marianne. It's difficult for me to catch the feeling behind the screaming. It becomes a very half-hearted attempt. I would very much like to try again, at another moment.

Notice that this work with the patient's scar tissue is probably the most direct application of the formula: "Feel, understand, let go" (see [19,25,31,32,33]). The gestalts are picked up directly from the tissue and processed on the spot. This implies the patient's full collaboration and easier, when one or several persons are present to give holding. Interestingly, the scars do often disappear subsequently.

As Søren dealt with the right side of my mouth, I get a lot of images from my paternal grandfather. When I, at the age of 2–4 years, visited my paternal grandparents along with my family on Sundays, he always had to finger my "lamb thigh", meaning that with his enormous fist he took hold of my right thigh and grasped very hard. Then he moved his hand higher and higher up to my lap. Everyone saw and heard it on Sundays.

From time to time I visited them. Every day they were taking a nap after dinner, and sometimes when my paternal grandmother slept, or pretended to be asleep, my paternal grandfather took me on his arm, as I myself went too slowly and took me to the basement. Down there he dragged and raped me. I feel that he just stood, then grasped my waist, my face turned to him, taking me up and down over him. It didn't take so long, and then he dragged me upstairs again, to my paternal grandmother who was waking. Then she would have me go with her to her work, to take me along in a very noisy machine hall without putting the ear protector on, telling there weren't any left and that I just should stop my ears. It was indescribably loud inside that hall, very loud indeed. At other times my paternal grandfather took me along outside and raped me out there. The basement episodes I do see most distinctly, and I remember the fear to go to the basement or just approaching it.

**Wednesday**

Session at the gestalt therapist [Anna received support from both our medical team and a gestalt therapist connected to the clinic during this period]:

I established contact with my strength. I must pay attention to my needs: I have to allow both to receive and to make demands towards Søren and towards my friends. In the future we shall establish contact between my inner child and my outer woman. Plan: I am in a psychological crisis, and the following three weeks shall pass by having a nice cozy time with relaxation, the colouring book, women's magazines, TV-serials, good food as well as sleep and rest.

**Sunday**

I can feel/sense that my dad was not allowed to have me for himself. He was the first, who committed an offence against me, but although I was his daughter, he did not own me. That is, my father could not have me for himself, this my paternal grandfather and my paternal uncle would not allow. My father should share me with them, otherwise they would punish him, hurt him badly. So, in order for my dad himself to be able to carry on his assaults he had to share me. I do not know which punishment they would adopt on him; maybe they would report him? That is unclear to me. My dad was very much and sincerely sorry about having to share me with them. He could hardly make himself to do it, anyhow not to begin with. I guess my dad was perfectly aware they would not be treating me like he did; I guess he knew they would be violent and brutal towards me. Nevertheless, my dad did that. Here come the episodes with my paternal uncle, which turned up while Søren was massaging the scars in the corners of my mouth:

The first time my dad took me along to my paternal uncle's shop, he let him take me to a coal black room, while he himself was passing the time chatting with the shop assistant, while his big brother was raping me, I believe I was about 4 years old.

The second time my uncle raped me was not in the same room, but in the shop itself. He placed my hands on a long wooden top, and then he had sex with me, apparently in my rectum. I am getting the sensation of being held round the throat, the chin and the mouth. It is as if I am actually screaming all I can; I am kicking, flapping my little legs. I am struggling, however his hand is so large that it covers throat, chin, and mouth as well and absolutely no sounds escape from me. My scream does never get out, but drowns in my throat. My legs are not allowed to run across the floor, escape from him; they are just hanging in the air, kicking.

The third time my uncle raped me is no doubt the worst and most sadistic one. He is lying in his bedroom and is waiting for me to go to the bathroom. I am at their place playing with my two cousins. He just snatches me, so! I am kicking and he holds his hand before my throat, chin and mouth and says to me: "*I bet you will get it*!" and then he places my hands on the bathroom tub, uses his other hand to grasp my waist, after which he has brutally sex with me, in the vagina and the rectum by turns. He shifts so quickly between the two that I get very muddled. I feel completely confused. The following sentences turn up: *He is cold, he is disgusting, he doesn't care, he is so violent*. After he has raped me, I cannot at all touch myself at the bottom. I cannot at all wash the blood and sperm away, because if I touch it, my vagina, my rectum and everything "at the bottom" will go to pieces or explode. The pain is unbearable and makes me completely dumb and paralysed. Before my uncle leaves me, he says: "*If you tell it to anybody, nobody will want to have you.*" The fourth time he raped me is also in their bathroom. Here he is sitting on the edge of the bath, and again he holds one hand before throat, chin and mouth, while his other hand is round my waist. I am turned away from him. He is penetrating my rectum. The fifth time, again in the bathroom, like the forth time, except that now he is sitting on the toilet instead of the edge of the bath.

The night between Friday and Saturday I experienced getting serial raped while sleeping. I wake up at the first rape, at 3:00 hours in the night. Then follow three subsequent rapes, totally four times. The sentence "*I have got nobody*" turns up. It is connected with my father lending me to my paternal grandfather and paternal uncle, and as my mum was like she was, I had got nobody to watch me. After these four times I feel like going to stop with the consultations with Søren, 100 percent. The note from the night I wrote en route reads: In a moment he is going to hurt me, very much indeed. A little later I get raped for the fifth time. I am so unhappy and have now got so terrible pains in the stomach and the abdomen that more than ever I am about to take a tranquillizer. Then come two more rapes; they are connected. The sentences: *It's really bad, I want to die, I want to go away, and I cannot have this*, turn up. And it is really bad. I call Søren, but he does not answer the phone. A little later follows a serial rape of five, so at last I got at total of 12 times. My last note from the night reads: "*Unbearable pain*".

A moment later something particular happens. The above sentences are striving in my head, so I do as follows: I clench both hands so hard that it actually feels as if I am locking them. I think: Now they cannot open again, and now it will stop. The sensation this time of being hidden is totally new. It is not like I have experienced it earlier. This time it is like towering walls of black armour plating are rising round me, and I think that absolutely nothing and nobody will be able to penetrate. Nobody. These high, black armour plates thus surround me and actually I am calming down remarkably quickly now, because nothing and nobody, indeed, can trespass on me. When waking up in the morning I am very unwell and I feel very sad. Furthermore I am angry with Søren, because he did not at all phone me on Friday, and I find he was mean. When getting hold of him, he says I shall get in touch with my gestalt therapist and talk with her, as she is my therapist now. I feel a confusion inside myself and think that maybe Søren is withdrawing from our therapy relationship. During the night I got a nightmare: Søren and I are going for a ride in the car. He starts scolding me, because of my irresponsibility and he is terribly angry with me. I wake up from the dream, just as he places his hands round my throat and is about to strangle me. I remember now: My mother asked once my father to kill me. I am asking our in the air: "*Why didn't they just kill me? Why didn't they kill me?*"

Then the next morning I talk with my gestalt therapist on the phone and we discuss my relationship with Søren; I have to find out what it is about, and if I can phone him and when.

**Tuesday**

Lat night I dreamt that Søren gave a tough message for me: “Spread your legs and get it over with!”, implicitly that the way ahead of me was the way through the pain from the numerous assaults. No time for pity!

So Sunday night my attitude was quite a bit different. I told myself that I could easily make it, that I was strong and that I shall be doing all right. Then I am on my stomach and let the thoughts come to me without struggling.

Assault: Just before I fall asleep, my father comes in my room. He penetrates me, my vagina, and it is quickly and non-violently. He is doing it almost gently and appears sad.

Assault: It is in the morning: my father is in the bathroom and I have to pee. My mother says: “Just go in there” and I do it. My father takes me to the shower cabinet and has sex with me. Again non-violently.

Abuse: My mother has taken me to the bathroom. Again I am not able to get rid of the stools; in any case not at the pace and at the moments my mother wants. She places me my hands and legs on the floor, and my behind up, like when she had to wipe me. She uses a spoon, and to start with it doesn’t go so far up because I tighten and work hard against this. Then she puts me with my stomach on her knees and starts. She says: “*You bet I’ll get it out*”. She keeps on digging, and this time she gets much deeper. However, there are no stools, and she keeps on saying she will get them out for sure. She is so eager to get something out and tries with the spoon to get in my rectum as deeply as possible. It is as if she has broke down, as if she is completely lost in the situation.

Assault: My paternal uncle. I guess it is in the bathroom while he is sitting on the bath. He penetrates my rectum; it’s most brutal, and I feel a huge pain. I feel it is something corresponding to a very hard pipe, which drives up and down in my rectum. It hurts far up, to the very end in front of the navel.

After all this *I feel rather exhausted*. Most of all I felt like being held close. Incidentally, I must add that I feel very comfortable with colouring in my recently purchased colouring book. I colour with crayons and I am perfectly aware this is as creative as I can possibly be, now that I am not more than four year old; so that is the way it should be.

**Yesterday night**

I am on the point of sleeping, and my mother is in my thoughts. Again I go into the episode from the previous night, the one where she told me she wanted to get it out for sure. Her whole energy in that situation is coal black and evil. Now I see her more as a sadist, so she appears; she didn’t cease while digging about in me; she just carried on, got ever deeper in my rectum as if she should get that out, no matter what – and she went on and on. She herself decided when to stop, and that didn’t depend on what and how much stools she got out of me; it was completely independent of this. Apparently, she didn’t even do it in order to get the stools out of me, because there weren’t any in that episode. She did it for the simple reason of doing it! A sadist, I guess, is an appropriate word indeed…

I also think of my nightmare from Friday night where she asked my father to cut me away and off his life. What is that woman’s agenda? It seems as if, only to a certain extent and maybe not at all, she is the victim in the home. The story has always been that she was the poor one, the one who was being oppressed. It rather seemed as if this story doesn’t really hold. I get the impression that my mother actually was ruling at home.

*Poor me*! I do now what I find most difficult and disgusting. I lay down on my back, in my bed. It’s terribly unpleasant, and also it doesn’t take long before I start crying, then sobbing. I’m sad. *It’s tough*!

Assault: my paternal uncle. Now comes the sensation of a piston up inside me. With the sensation follows the piston sound. I can hear it: “dunk, dunk, dunk” in tact. With my fingers on my stomach I feel exactly how far up the pain goes. It reaches up to the left of the navel; and then *I am gone*. It’s as if I am hearing the whirr of wings. I have got the impression, and more than that, that I am actually flying high up in the air. I am a bird, and I hear the wind blowing while *I am flying about up in the air*.

Again I lay down on my back, now with my fingers and legs spread. I think; I cannot do that, and then I do it nevertheless.

Assault: my paternal uncle. Again he knocks up inside me, brutally, and I feel again that the pain goes as far as up to the left of the navel. He simply hammers up in me, and the sound follows: “dunk, dunk, dunk”.

Assault: comes in series. Exactly the same situation, exactly the same contents. Just like that, again. It can only be my paternal uncle the second and third times!

Assault: my paternal uncle. I sense he presses and presses his too huge penis into my rectum. He presses and works hard, and slowly it gets deeper and deeper. It gets as high as to the left of my navel, and then I can sense that he has got an orgasm; he empties himself up there inside me. A second later I get an uncomfortable nausea. I get dizzy. His sperm is up inside me, and it feels as if it’s completely up in my throat; as if, if I vomited now, I would be able to vomit the sperm.

Rape: paternal uncle. The piston sensation, the sound: “dunk, dunk, dunk” and again pain till left side of the navel.

Rape: They press forward up in me like worms, it’s difficult indeed, but they just carry on like dry worms without any slime just waiting for the friction resistance to disappear. They slave away, persistently. Nausea again.

Now I lay down, my legs spread and my arms above the head, the fingers free. Terrible. Rape: paternal grandfather. This time I am completely paralysed with fear, totally paralysed, and I do not make a single movement. The pain goes to the left of the navel. I get the impression I am going to be killed if I do anything wrong, and I sense my paternal grandfather’s energy. Now I am gone. Now the wind is howling and I am out in a storm; there is snow everywhere. Am I on the Antartic? I guess so. I have to stop here, the sentences “*I get smashed up*” and “*I go to pieces*” appear.

Again I lay down on my stomach, and I can feel a huge pain. I am aching unbelievably, corresponding to the rectum and up to the left of the navel. Now I embrace myself, hold myself close, like I did just before all this happened tonight. I hold myself; it feels so nice and safe. It’s okay. In the evening, in the bath: I feel a warmth inside, it’s lust, I know. I don’t feel like touching myself at the bottom, so I massage and caress my breasts. I do it in a completely new way, and it’s really nice. It makes me glad.

**Tuesday night**:

Rape: father. I am supposed to sleep in the evening. He reaches halfway up towards the navel; it hurts, but he doesn’t do it so violently. He says to me: “I will …” and doesn’t complete the sentence. I guess he wanted to say: “be careful/do it carefully” or something like that.

Rape: father. He presses himself up in me. When up there, he makes stubborn pushes with intervals. He gets to the very bottom, but it isn’t violent. He takes me obliquely off the navel. I think “*It isn’t so bad*” and sense there might be some lust for my part.

Rape: paternal uncle. He slaves away, again with his huge hard pipe. I have got the impression it isn’t himself, rather an object; maybe a candle, it feels very thick. He reaches higher than the navel this time. Then it’s as if there is a shift, and now it’s he himself who is penetrating me. He enjoys this, and it’s the first time I hear him moaning. It takes much longer than usually and it isn’t violently now compared to earlier and with less hammering and beating. Now I get the sensation that my labias are forced apart, and that something is penetrating me. My vagina feels now huge and extended, like a hand; a whole hand is inside me. Then appears the sentence: “*I don’t want any more*”.

Then I consider how I will get my productive and hard working subconsciousness shut down …

As a solution I place myself in the doorway of an imaginary shop and say to the numerous “assaults” appearing and impatiently standing in front of the shop wanting to enter: “Sorry, the shop is closing now. I am sorry that I have to disappoint some of you. – You may come again tomorrow. Good night and sleep well everyone”. I didn’t want to make any of them cross, but wanted to admit that they are here and show them that it’s quite okay with me, however, that I am the one having the last word with respect to the shop’s opening hours! It was totally super cool that actually it did succeed! The shop closed indeed! It felt really good to wake up in the morning, on Wednesday; I got the sensation of controlling the situation. It was indescribably good!

Wednesday morning Søren came, because I had asked him to come and hold me close. So he did, and it was really nice. Wednesday evening in the bath: Again I feel the warmth and lust after having watched myself naked in the mirror. I observe my behind and the region round the arsehole. I find it has become prettier and can look at it and feel that I am more present in that region, especially round the arsehole. Its former flat appearance, the death, seems decreased. My breasts too have become prettier than they were earlier. Tonight I feel like touching myself at the bottom, and I am feeling myself, touching where it feels nice, and am thinking then: "*It's not so bad*"; consequently, there must have been a lust aspect with my father! It feels good what I am doing. Again I massage my breasts and think they are wonderful. Yes indeed! It's really nice to caress them and feel them as whole breasts. While I am doing this, something happens, which I regard as curious. I touch my breasts and at the same time I dream wearing my fine, blue summer dress; it makes me feel so super feminine. Curious! But it's great, and today I feel that my breasts are perfect and that I am gorgeous. I go to bed. Suddenly the sentence "*Nobody likes me*" turns up.

## Wednesday night

The sentences "*I hate her, she is nasty, she is disgusting, I loathe her*" turns up. Afterwards I stand on the shop's doorway. I tell everyone that I am very tired, angry and not at all in the mood for more. I say that I don't care whether they get cross, as I am the boss, and that I am really very tired and am going to close the shop. Full stop! They do not even protest, on the contrary it's as if they are mumbling they understand all right and wish me sweet dreams. Too wild! But I was simply done in; I was a totally flat battery yesterday as I went to bed at last.

## Thursday

The following sentences appear:
*It's odd*
*It's really curious*
*(I'm not good, am I?)*
*(I'm worthless, am I not?)*

## Friday

Yesterday afternoon I released myself "*I hate her*"; it was so extremely violent that I doubted whether I would stop crying again. I sensed how the earth disappeared under my feet, and I almost put myself across the table in order to hold on, have the connection. It was almost a revelation, I felt. "*I loathe her, she is nasty, she is disgusting*" – these sentences gave me such a heavy nausea that I hurried up to the kitchen to release above the sink. I was very close to vomiting. Two new sentences turned up: "*I'm good for nothing" and "I'm a failure*". As "I'm a failure" appeared, it was so terribly violent that it send me direct into space, without any earth connection, floating about between the planets. This made me dizzy. "I am out in the space". While at home and in bed, I think again of "*I hate her*" and "*I am a failure*". The sensation of being a failure, as well as my own self-hatred are the things that right now are worse than the rape. I write: "*I cannot be in myself*" and "*I want to leave*". Then follow:

Rape: my paternal uncle. The bathroom; he sits on the toilet. It goes so extremely fast, so quick up and down, in the vagina, I sense. So fast, so if I said a sound it would go like me sitting in a shopping trolley, driving across an uneven surface. The pain reaches the navel. Having my back to him, it's unclear to me whether he is holding his hand over my throat, chin and mouth, I guess so, and actually it doesn't bother me. I feel unbelievably like making fun of him, I really would like, while he is slaving away in me, puffing and blowing, to say that sound like when me sitting in a shopping trolley …I just think he is so ridiculous, and that it would be great fun to say that sound, as his wanting to hurt me so much would be such a marked contrast to my just making that sound, which is so funny indeed. That would thwart his sadistic agenda! When finished he said to me: "*You are worthless*"; and now I can feel that I understand no more. I did acknowledge it: "*I am worthless*".

4:35 – still awake, the garbage workers arrive outside

5:01 – still awake

5:30 – still awake

### Friday

Today something is different. I have got appetite again and have eaten quite a lot today, about 6–7 times! That's really good! Talked briefly on the phone with Søren tonight; he said that he is with me all the time, and I find I can feel that. He said to me that I have broken through! My eyes filled with tears, while he told me that, and it's also what I am feeling inside myself, I guess. Oh, this is fortunate! He said that I was not any longer in my dark side. Imagine I should break through! I am feeling really fine too! Actually, really fine indeed.

### Saturday

It was a wonderful evening yesterday; I just enjoyed staying at home together with myself. I watched the movie: "It's me talking" from I don't know when, and it made me laugh a lot. Old and entertaining it was great. I slept very well tonight and had two dreams.

1st dream: Where I was sexually together with a gorgeous girl; wonderful breasts, warm lap, and it was fantastic. I gave her quite a lot of compliments and enjoyed her a lot. At the end, in the dream, her labias broke; she was bleeding and showed me them. They were bloody, torn, and she looked badly knocked about. Then she said I should better be checked up for venereal diseases… (I found her gorgeous). 2nd dream: About me rescuing my sister from an evil man battering her.

Søren told me later, as I told him about the dream, that I was the one raping my sister. I can see that all right and told him about the time, where Marie (my sister) fell in love with me and I subsequently fantasized about wanting to throw her down on the bed and doing with her whatever I pleased. He said that it was good I did not get hold of her. I must agree. He also said that most likely I would not do that today.

### Saturday

A friend of mine talked to me about the relationship between Søren and me. That it is a unique doctor/patient relationship and I explained how he has been able to support me in very difficult times and open up for things that I had hidden, even from myself. She said she had been waiting for me to tell her about it. She said I could trust her. The amazing thing was that she did not condemn me at all; on the contrary she listened so carefully, respected me and acknowledged me fully. It was amazing how pure her intentions were. She respected me, acknowledged me and let me know that she wished me all the best, respectfully offering me that if I wanted I could discuss whatever I wanted with her. However, I feel I am doing fine now. As I said, it was an amazing conversation! At one point she began to cry; I told her that indeed I have a deeply based problem with trust and that in the future I will do my best towards a trusting friendship. Further she said that she knew I would succeed in this and she was looking forward to it, and meanwhile she was there for me. At a point, as we were discussing trust/lack of trust, I said: "I suppose you have guessed that I have been sexually abused", having hinted it earlier. She nodded affirmatively. She felt having to protect me/spare me any evil, including Søren, as she asked: "Could he think of anything sexual?" She mistrusted him, but I answered no.

Later, during the night, I begin processing. I am in a completely white room; there are no walls, no floor or ceiling. It is white and infinite. While I am there, two sentences appear: "*Nobody is there for me*" and "*I am miserable*". That is a true sentence, "Nobody is there for me", it fits fine on me, and I felt it so clearly that it made the sentence "*I am miserable*" appear. I release them during the night by means of the roll. "*I am miserable*" turns up again. At a quick pace follow: "I can do nothing, I know nothing, I am not good for nothing, I am always so foolish, I want to die".

Søren called me by phone, before I went to bed to ask, whether I was okay, and I answered that I will make it. However, while in bed with the wish to die and wishing for it so intensely, I became afraid of staying alone and called my ex-boyfriend, who came to hold me close the whole night. He stayed the following day until the afternoon, where I had an appointment with my gestalt therapist.

**Sunday night**
This is the night, where my boyfriend held me close.

**Sunday**
My mother and I are in our bathroom. She has been digging for a very long time, and suddenly she says: "*Now it's enough*!" She placed both her thumbs on my throat, in that small cavity and now really she wanted to kill me. She squeezed and the sentence *"She is going to kill me*" turned up. She carried on until I am lying unconscious on the bathroom floor. Maybe she thought I am dead now, or even a practical aspect turned up, where shall they put the body? How shall she make it seem an accident, when having been strangling me? I know, that I am not in the present time, and therefore I stay and wait.

**Thursday noon**
I took a bath, and it was a really unpleasant experience. I experienced I was sitting in our own bath together with my father; I was about six years old, and my father had sex with me. This time it was completely different. I was facing my father, looking at his chest and the relationship between us had changed. My sensation was that my father in a way accepted his lust for me and that he preferred having sex with me, rather than with my mother. I was his mistress; there was some equality, some acceptance. My father enjoyed me more than ever, and in a way I liked it too. In the bath at my friend's place, now I am suddenly heavily pregnant and am going to give birth. I think and say out aloud: "I cannot give birth to my child like that. – It will be a trauma for the baby." This comes up, while I am sitting in the position for a pelvic exam. I cannot manage to do it at all. I can now see the situation with my father in the car again: he hardy can have himself do it, to kill me, but my mother has ordered it. First he drives like a fool to subsequently stop the car in order to strangle me. The sentence "*He is going to kill me*" turns up. As I release it, also the physical aspect comes in. I cannot breathe, my trachea is choking, and I struggle for breath. I keep on struggling and manage to breathe before loosing consciousness. However, he did it so hard that I can still feel, half an hour later, how my trachea is choked and there is no free breath passage. Experiencing both my father and mother trying to kill me makes me indescribably sad. I find it difficult to believe that this is true, not in a denial way, but in the naïve way, which is my way of surviving my parents' evil.

One one side
On the other side
A repair: Black side:
Naive, simple-minded towards
Believe people are worse than they actually are
People's evil intentions
*He and she are going to kill me*
When I told Søren on the phone he suggested I work at these disequilibria with my gestalt therapist.

**Friday evening**
The sentence "*There is no room for me*" comes to me. The sentence "*I am nothing*" re-appeared.

**The night from Saturday to Sunday**
Dream: I am in a garden together with two girl friends and another girl. We are two couples; we are chatting, going for a walk and having fun. At a window a women with her companion are making fun of the four of us and find we are very much "out of fashion" as lesbians, it's just so "out". They are making fun of us. Up in the apartment, the home companion is going to bed, and the women find that her companion is foolish and narrow-minded. As she looks uphill she sees her own father observing her from a big balcony. She herself is in her room, and he is looking down at her. Suddenly I am in her apartment, and we are lying together naked in her bed. It does not bother her that her father is disappointed that she is not giving him grandchildren. She wants to stay together with me and does not care about him. I am enjoying her; I am enjoying looking at her, touching her. She is just gorgeous, like a dream.

### Sunday

I have got a particular sensation, I am in a special situation. Never before have I been experiencing so massive, so intense, so deep a gestalt. "*She is going to kill me", "He is going to kill me", "They are going to kill me*" are some really cruel gestalts. A moment ago, as I was talking with Søren on the phone, the sentence, which also came to me at the movie yesterday "It's really serious" turned up. It contributed towards making the situation enormously heavy to me.

Søren said that the decisions we are making become our wishes as well, which – if not carried out at that time - will remain our wishes, which we want to carry out. Consequently, I wished to be killed, and this makes me attract people who wants to kill me. I attract the perpetrator. Right now I am feeling sorry for myself. Yuck, do release that too! I am releasing: "*It's really serious, I feel sorry for myself, she is going to kill me, he is going to kill me, they are going to kill me*". Søren told me that the universe is gracious and allows us all to commit mistakes once.

### After supper, on Sunday:

1) Rape: my paternal uncle in the bathroom. He holds me in a new and unpleasant manner, I almost hang, my head down, while he is shifting between the holes. It aches up to the navel; I am dizzy and have got nausea. Meanwhile I think that I will be going to the sink afterwards and vomit. He gets his orgasm in my mouth, and next I am squatting at the sink. The sentence "*I feel so bad*" comes up. At a point I am out of myself; I am going to faint and this because the pain is unbearable. I don't know where I am; it's dark. I am older, maybe 5–6 years.

2) Rape: my paternal uncle. The pain is completely unbearable. He is hammering and humping like a fool. It hurts indescribably. At a point he hits something up inside me, and it almost says "klonk". What does he hit? It hurts so much! I am not able to scream, he is holding me. I cry from pain.

3) Ill-treatment: my mother in the bathroom. She is digging me out; huge pain. I almost jump every time she digs inside me. The movement is totally different than that of the men's; she gets much more aggressive and is everywhere inside me. I can feel how the spoon is scraping up my rectum. Now I go to the toilet, have diarrhoea, and I tell them: "It's fine you came, and now you must leave". Afterwards I am getting better.

My ex-boyfriend came in the afternoon and while we are doing the dishes, I told him that both my father and mother have tried to kill me, and that several of my family members have abused and raped me. He becomes terribly angry, because it is unbelievable that I am telling him this and that he is here, while I am reliving the three above episodes. I think that is why I relived such a huge pain; he loves me and backed me. I appreciate that. He is an invaluable support for me. I guess his purpose is: "I am good, warm and affectionate". The sentence "*I am miserable*" appeared during the conversation with Søren.

### Monday

I am useless, I can nothing, I am so foolish. I feel like lying like a little baby in a cradle, being rocked, fed, changed. I want to be taken off. To be nothing and yet to be everything for myself, that makes me crazy. The black painting with the light stroke. You must paint it, Søren said. It represented how I was inside my mother's womb; she was coal-black, then I came and was lying in her uterus like a snow-white stroke.

### Friday

I told him how my father had often been saying that my mother was a schizophrenic. Søren said that I had taken that in, and that my sentence was "She is a schizophrenic". That sentence did hardly want to leave my fingers! I had been taking it in so well that I had to make an effort, and I got easily short of breath when having to release it.

Earlier I had been talking on the phone with a friend, who was also grumpy, and I had told her that it felt as if I was at the bottom of a mash; muddy and turbid. After having let go of “She is a schizophrenic” Søren said I should let go of “I am a schizophrenic”; then indeed I started struggling. My defence had been moved into position; I told him that the sentence was not like that, and I resisted a lot. Of course, it proved to be the completely right sentence, and probably much more difficult to release than the earlier one. On releasing it, something fantastic happened. Suddenly it turned completely bright behind my closed eyes, and then followed a light. It became so wonderful inside me; it felt so indescribably well to be inside myself. Fantastic! And then I could say: I WAS a schizophrenic.

My friend was still cross and lovely at the same time. I briefed her on Monday’s processing with Søren. I told her that I had been a schizophrenic, and it was so indescribably nice to share this with her. She did not become frightened or run away from me. She could easily have done that.

Wednesday evening I am dining out with my best friend and later we watched a movie at his place. It was a very tough movie on abuse. Afterwards I told him about Monday’s episode. Damned if I did not tell him that I had been a schizophrenic!!! Well, I really did so, and while telling him I realized that his purpose of life was probably: I do contain.

## Thursday

The following sentences turn up: *I am getting ill now, I am useless, I don’t care any more, let me die, you don’t care, I am ashamed, you must leave me alone, they must leave me alone, I am dying now, I am very scared.* Suddenly I feel I get completely cleared inside of any struggle, discomfort and unpleasant feelings whatsoever. I suspect I jumped out of myself; how can it be so silent, quiet and nice inside myself? I simply cannot at all see or sense where I should have jumped. Therefore I think I still was in myself and that the peace was due to me having won the last and decisive battle against the ultimate evil! Then I got so sleepy that I had to lie down on the floor and rest for about half an hour. Next I sat up again and started releasing. I went into them all, and “I am ashamed” and “I am very scared” (of what might happen to me; to get insane, etc.) were particularly tough. I sense, like I have been sensing the whole day that I have not even got a façade at all; that there is nothing I can use, I am completely nude. Anna was the borderline/schizophrenic girl, and now that I have released that, I feel I have got almost no personality! I have to define myself on a new, clean slate and from the beginning. Who am I? This appears a relevant question now… At home I take out the telephone. I do not know what to say to people, and I do not want to talk with Søren.

My girlfriend is visiting me, and I tell her sincerely how I have experienced not respecting her and that I want to apologize for that. I saw my lack of respect towards her expressed, because I never phoned her and took too long to reply to her messages on the answering machine, the same for her e-mails, and too many times I cancelled our appointments. Then I said that for this reason I find I have not been a friend, nor am I motivated/feel like being her friend, and that we should stop it here. Furthermore I tell her that I have the feeling of breaking a pattern of hers about always giving and not getting or wanting to receive anything in return. I say I do not want to help her maintaining that pattern of hers; neither she nor I deserve that. Although I had got red rings under my eyes after the day’s massive, heartrending crying, I was feeling remarkably well as she visited me. What I said and did was feeling so right, and actually it was the first thing I felt right as an ex-schizophrenic, right indeed. It made me feel indescribably well to be so sincere towards her and conclude a malfunctioning friendship. I found it was really smart for my part.

In the evening the shame come up in me again and “*I am very scared*” comes also up again, and I let go of the sentence. However, it is as if I did not fully succeed in getting rid of the shame. Later I talk with my friend; she is tidying up too, and next we talk about my day’s events. I told her then that I could feel in myself I had to go away for some days, to a strange place, where nobody knows me in order to be and get closer to an answer as to who I am. It feels right. I told her I wanted to go to the youth hostel in the north. She approved and said she found it felt right too. Furthermore we talked about my family name, because I was ashamed of it due to my father’s family, and now I also feel Anna does not match. She asked me to think about some other names, about how I found them, and about what I find is matching.

We discuss several options and it sounds great! Really beautiful and it feels right too. Yes it feels good, indeed.

## Thursday night

I wake early in the morning. Friday morning; my heart is galloping, and I can feel I am afraid that Søren might get angry with me, about my reporting sick, about perhaps me not having contacted him at all for several days or him not being able to contact me. I am scared. At 5:30 am I phone him and leave a message that I am ill. Fall asleep until about 7 am and again sleep until 10 am. Then I start getting ready to leave for the north. It feels so right and as a necessity to leave for a couple of days. Now it is as if even the apartment makes me frustrated and I guess maintains me in a wrong image of myself. I was very much in a hurry to leave home today. In the train on my way up I got unwell. Thought again of Søren's probable anger; will he feel that I let him down, or will he think "All right, this was Anna, she was a schizophrenic; I am so happy to have got rid of her"? Now I am able to concentrate on the important things.

The sentence "*I'm not important*" comes up. Released, hmmm. I don't know about Søren; I'll see. From one train I transferred to a little train to go further and when getting off, I caught sight of the wonderful woods. My eyes filled with tears, and for a short while I was enjoying getting overwhelmed by nature's beauty. What a gift, so wonderful it is, I am thinking. I find the youth hostel and have got about one hour and a half before checking in. I go down to the sea; it smells good of seaweed, it's windy. I sit a moment on a big stone and then can feel how the beautiful forest is pulling me in. I go up there again and get completely reabsorbed and warm inside by its beauty, how differentiated it appears in all its multicoloured autumnal glory and how spiced it smells. I take in all its beauty and have got to stop many times, just to enjoy the sight and the smell. I have got the feeling of being at the right place and of belonging to. I walk about and finally reach the top, up in the woods, and find (am pulled by it) a very fine spot on the coloured foliage carpet, among the woods' probably oldest trees. I look at them, smiling and then sit down among them. From there I have got the view over the woods, a few houses and the sea. A perfect spot, indeed! I'm feeling so fine there! I gathered various things from the woods to decorate my room, and by the way I am enjoying to the full nature's abundant and formidable grace, grandiosity and beauty. Perfect spot!

On getting my room I settle and lie down to rest/sleep. I'm up again in the evening and go down to eat in an Italian restaurant close by. From there I call my friend. She praised me a lot for having left and said that it is so brave and strong of me, just to sit down in the train and go and stay up there by myself. It also feels tough, actually. It has been hard as well. It is difficult to be in me, especially when having to relate to other people; for what am I going to say and what am I going to do? I know it will come little by little, that it just does not come at once, and that most likely a long period with a lot of nudity is awaiting me. I will cope with that too. Afterwards I walked down to the shore, in the woods as well as up on the road. I could sense that I have almost got rid of my strained relation to darkness. I could easily be in darkness at the seashore, walk through the dark woods down to the sea, and I was feeling fine. It is not completely okay, but I believe it will become. At the youth hostel I am writing in my bed. I have written 18 pages now, and it does me good to have got this whole story down on paper. It is always a relief for me to be writing about it, as if it does not any longer haunt me in the same way; thereby I get control of it. The image of a chicken, its head just broken through the shell, peeping at the world for the first time; like this I am feeling today. Now I am looking forwards like a new-born baby-bird; I see the world once more.

## Saturday

After a nice bath I dressed and got ready to go out enjoying the nature. To begin with I must say that the sun has been shining on me the whole day; so beautiful and fine that I could not help saying hallo, while warming me on the outside and on the inside. This grew into many greetings. I was down near a Castle and was sitting at the end of the avenue with the sun on my face. The place was really beautiful and minimalistic. I proceeded a bit towards the city, but it was not the right way; too many cars, houses and first and foremost, too much noise. Therefore I went back and followed the beach with my recently purchased goods in the bag: chips and white bread, uhmmm. How great an outing it had been! I could sense how I got calmed and at the same time boosted from listening to the roar of the waves. I sat down on a big stone, on the cushion I had brought along, enjoyed the sound of the water and shuddered slightly at the warm sunbeams. While sitting there I thought of it once again: that I am an unwritten leaf and that right now I am exceptionally lucky, because I have got the chance to shape my life and myself like I want it. Now I can cultivate the capacities, skills and qualities I greatly prefer to possess, and this without the usual, rotten wreckage in tow, which could prevent me from doing it. There is a huge opportunity right now! I was also thinking this means that now I will actually be capable of getting/achieving ANYTHING I want. I just have to set about getting/achieving it. I walked about 6 km, along the beach and walked back via the woods. I thought of the song “The woods around the country are turning yellow now” and changed the title into “The woods around the country are glowing now” because I find that was what they actually did. The woods are unbelievably beautiful right now, in fact my eyes filled with tears. It is indeed a huge gift for me to be up here in the beautiful nature. It was, no doubt, the completely right impulse to follow! As I returned I began to paint/colour a bit in the colouring book. However, it did not really mean anything to me though, so I did not finish the drawing. It was so boring … Then I laid down to rest. I dreamed a little, but as I did not manage to maintain the dream, I forgot it again.

Now I have been taking a bath and I want to read a little, before going to sleep again. Tomorrow I shall check out in the morning. I am curious to know whether I will wake up, having got no watch. What I have been experiencing has been right: To stay in the pleasure: going for a walk today, giving myself in to the pleasure; not holding back at all and being able to stay in it. To give myself for: the anger. The same principle as for the pleasure: not holding back, being one hundred percent in it. - This is to be alive!

## Saturday night

I twist about miserably in my bed, sweat as if I had got a very high temperature. The anger is huge, and while lying here I am full of it. I beat the mattress, swear, snub them, and then begin projecting anger onto Søren. I get angry with him at the way he treated me this week; the rough way mixed with an apparent indifference as to the way he had been reflecting me. Then the suicide thoughts appear:

pistol: too much mess and too traumatic for those who find me cut my throat: same thing as with the pistol cut the wrists: then I shall suffer too long liquidation: I could pay someone to do it; this seems the best, until I am thinking of:

overdose: which would be much better. A second later I think that I would probably not know the correct dosage, but would brain-damage myself and end as a vegetable, dribbling and not even being able to communicate to people that they must kill me. Then Søren would call me, hold me close, and this would be the ultimate hell; me not being able to communicate, only dribble. Now I cry and am totally miserable. I still sweat fever. I think then: Stop – just be quiet. One day at the time. I say aloud: “I bring life and joy. I bring life. I bring life.” quite a lot of times, and this calms me; this slowly makes me relax. [Anna is here assuming responsibility for her own existence at the most deep level; she is facing the need to choose to live or to die, accepting life on its own conditions or not accepting it. This is really the deepest level of existential choice for any human being: do you want to live or do you want to die? And it is a strictly personal question; nobody can really help you out here, you need to solve this for yourself, as Anna instinctively did].

## Monday

At long last I had a decent conversation with Søren. I had hurt his feelings, made him sad. He said I did it to create a distance between us. I told him that I was fond of him. He thanked and finished the conversation saying he was fond of me too.

Subjects: detachment, independence.

I slept very bad tonight, Søren and our understanding dialogue about “what did happen” the last few days being constantly in my thoughts. I even wrote a poem while shifting about restlessly.

**Tuesday**
I was at my gestalt therapist today. Further I am thinking that if I play my cards well I can end as something big. With my story, my intuitive intelligence and my courage I think I can become an entirely tough therapist. Watch me!

Later I talked with Søren; he was making fun of me and said I would soon be able to take my gestalt therapist in therapy. It was funny said, and I must admit that later on I will not forget her face, while telling her how I had been experiencing my therapy. Not only was she gaping, she also realized that she was facing a very intelligent girl, who had just discovered how intelligent she was. An educational experience, indeed!

Let me finish here by mentioning that my personal development will no doubt carry on. I have been releasing so amazingly much insecurity. Never before have I been feeling so confident that everything will turn out all right. I find I keep on getting ever more gorgeous, and I am sure I shall get the best boyfriend in the whole world. I am in the process of being quite happy; I am not miserable any more. I am convinced I shall become entirely happy.

Signed “Anna”

## DISCUSSION

The major problem of working with incest or childhood sexual abuse patients using the four cardinal steps of holistic medicine

- Love
- Trust
- Holding
- Processing is the extreme degree of closeness and intimacy this process involves. In this case, representing the worst possible abuse over a long period in childhood, there is hardly a feeling that has not been felt and hardly a spot on the patient’s body that has not been touched by the abuse.

To do this in an ethical way, a strict ethical code must be followed[1,25,26,31,32,33], but actually more than this, a deep ethical contemplation is needed to adapt existent ethical theory to the holistic medical clinic. Today, there are tree main lines of ethics:

- *Normative ethics*, setting the fundamental standards, is a most important subject for medicine and the physician, and when it comes to holistic medicine ethics, it is not only a means for securing that the patient is not suffering any harm — the first Hippocratic demand to the physician was: do no harm — but it is also a primary condition for the holistic medicine to work in the clinic. The reason for this is that only a totally focused intent to help the patient and to be at his or her service can make the changes in the patient’s existence and consciousness correlated to the holistic process of healing. So, in a way, the ethical perspective, the goodwill, and intention of being at service for the patient is what helps. In a way, it is a much more simple task in biomedicine when it comes to quality control because if the examination is done correctly, the diagnosis is right, and the medication comes with advice according to the book, then the “pneumonia” is optimally treated. In consciousness-based medicine, it is not so simple. If you meet a patient without respect, you lose the trust of the patient, and the cure will not work. It is as simple as that. So if you are not intending respect, you will fail. You will also fail if you are not aware, careful, accepting, or acknowledging the true nature of the patient. And you will fail if you are not able to support your patient in feeling, understanding, or in letting go of negative beliefs. So actually, holistic medicine is an art based on ethics, while to a large extent biomedicine is a skill based on mental knowledge and intelligence. How does the ethics of holistic medicine fit to ethical theory? This is not a simple question to answer.

- *Teleological ethical theory* is one of the philosophical main ethical positions focused on consequences and claims that what is obtained by an act is the essential. The *deontological position*, on the other hand, is about duty and claims that the intention is what really counts. It is very interesting that because we are dealing, in a way, directly with consciousness and experiences, the teleological and the deontological position are both true at the same time, in holistic medicine. The intention creates the result and actually the result mirrors the intent. This is fairly true when it comes to holistic healing, but it is not true in absolute terms. If I intend to help a patient mortally ill with cancer and I prolong the suffering without creating any good for the patient, I have had good intentions, but my results were bad. Sometimes, this problem is solved by asking the patient what he or she wants, but the duty-ethical position is that deep inside, every life has the wish to live; the patient might take the position of the ego, not his true self, and so he is tricked and the physician, following this patient's verbally expressed choices, is tricked as well. Therefore, suicide is not a good idea. Interestingly, the two different approaches to ethics can be seen as either a preference for power — what can be obtained — or a preference for love and purpose. But there seems to be one more fundamental dimension in human existence and that is gender, or sexuality, and the balance between these two.
- The *feministic ethics* arise from the third position, claiming that the two genders carry two different sets of values. It is necessary to be conscious of these two very different sets of human values, for if you happen to exclude one set, you will pull or push reality out of balance, and the intended value cannot be realized. In holistic medicine, this argument seems to be extremely important in that the classical female values — represented by the qualities of holding — are to be complemented by the set of classical male values — represented by the qualities of processing. It is important to understand that these qualities are not bound to sex or gender in a simple way and the holistic physician can provide both male and female qualities. He can be "the good mother" for a patient who never had one, to heal the early wounds of failure, scolding, or neglect. A core idea for Jungian-inspired holistic medicine is that everybody contains both the male and the female, and only if both parts are understood, accepted, and integrated, can the human being — patient as well as physician — be a whole, healthy, talented, and happy person. Interestingly, many of the "bad" things physicians have done in the best of intentions[34], like clitoridectomia as a treatment for "nymphomania", can be understood as a result of the feminine values not being sufficiently represented.

Clinical work with incest victims and other sexually abused patients have forced us to put much more focus on gender and sexuality, as many problems are found here symptomatically, and as many more arise from the lack of joy and pleasure coming from the patient not being in balance on the male-female axis[35]. Our ethical position is now a balanced view between these three extreme ethical positions. We must have the best of intentions, but we must also look carefully at our results, not harming our patients. We must be loving and powerful, but we cannot ignore the dimension of gender and sexuality, how impractical and hard-to-integrate-with-hard-medical-science such dimensions might appear.

The holistic physician is, like any other physician in any medical paradigm, free to choose any of the three philosophical positions of ethics, but there may be only one clear ethical position that will be of most benefit to the patient. To explore this different field of ethics is an important part of the job to be done in future papers on holistic medical ethics. Some of the main arguments of Emanuel Kant, John Stuart Mill, and the feministic thinkers must be discussed in this connection.

There is also the problem of normative ethics: it varies from culture to culture, so what is the universal applicability of clinical holistic medicine? For example, the use of touch and acupressure through the vagina are basically unacceptable in the Asian cultures. The universal applicability seems to be that loving care, trust, holding, and processing can be a part of any treatment, anywhere on the planet. The degree of intimacy must always be adjusted to the actual culture and the local legal requirements. As some the procedures involve close physical contact between the therapist and the patient, how can we ensure that the therapist is not exploiting the patient in the process? This is one of the most important questions, but sexual exploitation is fairly simple to avoid as sexual behavior between the physician and his patient has been forbidden ever since Hippocrates. But there are other more subtle, emotional kinds of exploitation, i.e., the physician wanting confirmation or admiration from his patients, treating them in ways to obtain that. The only way to avoid this is by strict supervision; the supervisor is much more likely to observe power-games and unwanted transference and countertransference than the physician or therapist him- or her self.

Another important question is if the more physically intimate procedures are consistent with the code of practice of the mainstream medical and counseling associations in the Western world. Many counseling associations in psychiatry, psychology, and cognitive therapy prohibit counselors to have physical contact with clients of the opposite sex and touching the clients' genitals is strictly prohibited. On the other hand, there are many organizations of body workers that stress the importance of intimate physical contact in therapy. As the clinical holistic medicine toolbox is not yet endorsed by the mainstream, we need to alert the readers that the related procedures are more or less controversial in nature, in all therapeutic fields where body work is not usual.

Finally, what are the limitations of using verbal reports to support the claim that the intervention is successful? It is important to understand that both the therapist and the patient must feel that the therapy has been successful in the end. Such verbal reports do not necessarily prove that the therapy is going well, but at all times, the therapist must follow the patient from an objective position and evaluate how the presented written material from the patient can be understood. If the patient considers life worth living, the therapist should evaluate carefully if the patient has any tendency to commit suicide. Very often, the written words in the patient's diary are expressing strong feelings and deep existential thoughts that in another context, i.e., a suicide letter from a desperate teenager, would mean the highest alert. In therapy, where the feelings are expected and the process is in full control, even expressed thoughts of committing suicide almost never indicate that the patient has such actual plans. The diary is simply about containing and integrating the unbearable feelings from the past.

## CONCLUSION

Anna's story showed us what it takes to heal from extreme severe sexual abuse for years of her early childhood. It is really amazing that she managed to continue her process of healing even when the emotional pain got worse. The secret was the environment with so many people that wanted to help her, and her courage to open up and accept the help she needed. Anna's life would undoubtedly have been a life in and out of mental institutions if she had not done this great work of healing. It is important to stress that it was Anna who did the work herself; she wrote hundreds of pages of diary, she worked for many hundred of hours on letting go of hundreds upon hundreds of negative decisions taken in the painful events of abuse.

The role of the physician is as the good and patient father who gives the patient the love, care, awareness, respect, acknowledgment, and acceptance that she needs so badly, because she never received it from her own father or mother, who just did all the wrong things in one long series of extremely painful and systematic traumas.

What is to be learned from this story is that almost any patient can heal when given the proper support. We believe that the support must be holistic in its approach to mobilize the necessary resources for healing. The holistic process theory of healing gives a good model of this healing; a source to unlimited resources seems to be the recovery of the purpose of life[29,36,37,38,39,40,41,42] and working with this in therapy seems to be what motivated Anna and kept her going in spite of all odds.

We are honored to have worked with Anna and we send her all our best wishes for the future, which we actually expect to be excellent. Because of all the existential pain, where you take responsibility will end as learning and insight, and give you wisdom to love, forgive, and lead a rich, full, and successful life. We believe that Anna will get much more out of life than a normal girl who never was abused and never healed her existence from the most fundamental level. The lesson for us all is to learn from Anna's history, which she so generously has shared with us. Life is really nice, strong, and intrinsically valuable that even the darkest of events and the most evil of men cannot really destroy it. As long as the body is intact, the person inside can heal, but only in love.

Most importantly, Anna and many patients after her have demonstrated, that in spite of the depth of the existential crisis, holistic existential therapy seems to support existential responsibility so well, that the therapy is very safe for the patients. With more than 500 patients treated with holistic existential therapy at the Research Clinic for Holistic Medicine in Copenhagen, we have never experienced a single patient, who has tried to commit suicide during or after therapy. Neither have we seen a patient, who developed a mental illness provoked by the treatments, because the existential crisis is always temporary. This implies that holistic existential therapy is safer than standard biomedical psychiatric treatment.

## ACKNOWLEDGMENTS

This study was supported by grants from IMK Almene Fond. The quality of life research was originally approved by the Copenhagen Scientific Ethical Committee under number (KF)V.100.2123/91.

## BIOSKETCHES

**Søren Ventegodt, MD,** is the director of the Nordic School of Holistic Health and Quality of Life Research Center in Copenhagen, Denmark. He is also responsible for a Clinical Research Clinic for Holistic Medicine in Copenhagen and is a popular speaker throughout Scandinavia. He has published numerous scientific or popular articles and a number of books on holistic medicine, quality of life, and quality of working life. His most important scientific contributions are the comprehensive SEQOL questionnaire, the very short QOL5 questionnaire, the integrated QOL theory, the holistic process theory, the life mission theory, and the ongoing Danish Quality of Life Research Survey, 1991–94 in cooperation with the University Hospital of Copenhagen and the late professor of pediatrics, Bengt Zachau-Christiansen, MD, PhD. E-mail: ventegodt@livskvalitet.org. Website: www.livskvalitet.org

**Birgitte Clausen, RN,** leader of a secluded ward for people with dementia. Teacher at Nordic School of Holistic Medicine. Dementia coordinator and consultant in Aarhus, Denmark with more than 20 years of experience in adult psychiatry and dementia. During the last 4 years, she has worked together with Søren Ventegodt on the "QOL as medicine project" and on developing existential holistic group therapy. Through her articles and lectures, she has disseminated "The New Culture of Dementia" in both Denmark and Japan. She has participated in the production of several videos to order to inspire relatives, medical staff, and volunteers to understand people with dementia. This project has been granted financial support from the Ministry of Social Affairs in Denmark. She has conducted the first Danish investigation on the effect of sufficient daylight on the human spirit, activity level, and biological rhythm. E-mail: villahvide@mail.dk

**Joav Merrick, MD, DMSc,** is professor of child health and human development affiliated with the Center for Multidisciplinary Research in Aging, Zusman Child Development Center, Division of Pediatrics and Community Health at the Ben Gurion University, Beer-Sheva, Israel; the medical director of the Division for Mental Retardation, Ministry of Social Affairs, Jerusalem; and the founder and director of the National Institute of Child Health and Human Development. Dr. Merrick has numerous publications in the field of child health and human development, rehabilitation, intellectual disability, disability, health, welfare, abuse, advocacy, quality of life, and prevention and received the Peter Sabroe Child Award for outstanding work on behalf of Danish Children in 1985 and the International LEGO-Prize ("The Children's Nobel Prize") for an extraordinary contribution towards improvement in child welfare and well being in 1987. E-mail: jmerrick@internet-zahav.net. Website: www.nichd-israel.com
